# Supplementary material for: Community-based newborn care intervention fidelity and its implementation drivers in South Wollo Zone, North-east Ethiopia
Source: PLOS Glob Public Health. 2023 Aug 11;3(8):e0001168. doi: 10.1371/journal.pgph.0001168 (PMC10420373; doi:10.1371/journal.pgph.0001168)
Supplement: Attachment — Submitted filename: Response to reviewere.docx [file pgph.0001168.s001.docx]

January 24, 2023

Shela Hirani, PhD, IBCLC, RN
Academic Editor
PLOS Global Public Health

**Re**: Community-based newborn care intervention fidelity and its implementation drivers in South Wollo Zone, North-east Ethiopia (PGPH-D-22-01354)

Thank you for the constructive comments for the review of our manuscript. We appreciate the time and effort the editor and reviewers have dedicated in reviewing our manuscript. We have revised the manuscript as per the suggestion provided and highlighted those changes in blue in the revised manuscript. We have also improved the quality of written English. . We believe the manuscript is now improved and suitable for publication at PLOS Global Public Health journal. We look forward hearing from you in due course.

Sincerely,

Asressie Molla Tessema (PhD), On behalf of all authors

**Here are the point-by-point response to the editor’s and reviewers’ comments and concerns.**

**Editor’s comment**

**Comment 1***: Kindly incorporate the suggested amendments. Revised manuscript must be in accordance to the journal’s guidelines*

**Authors’ response**: We thank you very much for sharing your concerns. All the raised suggestions have been addressed carefully including adherence to journal’s guideline.

**Comment 2**: *This paper requires language editing from an English language editor*

**Authors’ response**: The writing language has been carefully edited as per the comment

**Comment 3**:

- *A rebuttal letter that responds to each point rose by the editor and reviewer(s). You should upload this letter as a separate file labeled 'Response to Reviewers'.*
- *A marked-up copy of your manuscript that highlights changes made to the original version. You should upload this as a separate file labeled 'Revised Manuscript with Track Changes'.*
- *An unmarked version of your revised paper without tracked changes. You should upload this as a separate file labeled 'Manuscript'.*

**Authors’ response**: The rebuttal letter, a marked up copy and unmarked version of the manuscript are prepared and uploaded through the portal.

**Comment 4**: *Please send a completed 'Competing Interests' statement; including any COIs declared by your co-authors. If you have no competing interests to declare, please state "The authors have declared that no competing interests exist". Otherwise please declare all competing interests beginning with the statement "I have read the journal's policy and the authors of this manuscript have the following competing interests:"*

**Authors’ response**: Thank you for the inquiry. Though there exists competing interests statement, we have re-written and corrected

**Comment 5**: *In the online submission form, you indicated that "The data for this manuscript preparation is found in the hands of corresponding author and can be accessed without restriction up on request". All PLOS journals now require all data underlying the findings described in their manuscript to be freely available to other researchers, either 1. In a public repository 2. Within the manuscript itself or 3. Uploaded as supplementary information.*

**Authors’ response**: Accepted and we have uploaded the dataset as a supporting information-compressed/zip file achieve.

**Reviewers’ comment**

**Reviewer one**

**Comment** 1: *Abbreviation should be written in expanded form when they are used for the first time. Eg, MNCH*
**Authors’ response**: Thank you for pointing this abbreviation. Accepted and corrected as on page 2.

**Comment** 2: *The authors didn’t mention the source of the formula they have used to compute overall frequency and content. What is their base to do so?*

**Authors’ response:** Thank you for this clarity inquiry. The authors have modified and utilized the conceptualization of fidelity from Carroll et al.^1^, Hasson et al.^2^ and Pérez et al.^3^ as well as computational approaches from Leegwater et al.^4^, Fernando C Wehrmeister et al.^5^, Wehrmeister et al.^6^ and Hernica et. al.^7^ as the base for computing coverage, frequency and fidelity.

1.	Carroll C, Patterson M, Wood S, Booth A, Rick J, Balain S. A conceptual framework for implementation fidelity. Implement Sci [Internet]. 2007 Dec 30;2(1):40. Available from: http://implementationscience.biomedcentral.com/articles/10.1186/1748-5908-2-40

2.	Hasson H, Blomberg S, Dunér A. Fidelity and moderating factors in complex interventions : a case study of a continuum of care program for frail elderly people in health and social care. Implement Sci [Internet]. 2012;7(1):23. Available from: http://www.implementationscience.com/content/7/1/23

3.	Pérez D, Stuyft P Van Der, Zabala C, Castro M, Lefèvre P. A modified theoretical framework to assess implementation fidelity of adaptive public health interventions. Implement Sci [Internet]. 2016;1–11. Available from: http://dx.doi.org/10.1186/s13012-016-0457-8

4.	Leegwater A, Wong W, Avila C. A concise, health service coverage index for monitoring progress towards universal health coverage. BMC Health Serv Res [Internet]. 2015;15(1):1–8. Available from: http://dx.doi.org/10.1186/s12913-015-0859-3

5.	Wehrmeister FC, Restrepo-Mendez MC, Franca GVA, Victora CG, Barros AJD. Summary indices for monitoring universal coverage in maternal and child health care. Bull World Health Organ. 2016;94(12):903–12.

6.	Wehrmeister FC, Barros AJD, Hosseinpoor AR, Boerma T, Victora CG. Measuring universal health coverage in reproductive, maternal, newborn and child health: An update of the composite coverage index. PLoS One [Internet]. 2020;15(4):1–10. Available from: http://dx.doi.org/10.1371/journal.pone.0232350

7.	Hernica C.W. De Vet, Caroline B. Terwee LBM and DLK. Measurement in medicine. Vol. 4, Cambridge. 2011. 88–100 p.

**Comment** 3: *No details are given about the validity and reliability of the translated tools used*

**Authors’ response:** Thank you for indicating this important concept. Accepted and corrected as in page 4.

**Comment** 3: *In socio-demographic characteristics it is not clear why the denominator is varied. For eg. 894,893, 888, is there data missing? The way the result described lacks uniformity throughout the document. For some variables (180 (20.04%), 246/260 (94.62%), and Thirteen (81.3%)*

**Authors’ response**: We appreciate your concern. Due to some variables' non-response, the denominator fluctuates from one variable to the next. Additionally, there were two categories of participants in the dataset we used: maternity clients (client level) and provider levels (Health extension workers). As a result, the percentage has been calculated using the appropriate denominators that take into account the response and participants.

**Comment** 3: *The major problem in multiple linear regressions is multicollinearity, how did you address this?*

**Authors’ response**: As a collinearity diagnostic, the variable inflation factor (VIF> 10) and tolerance (0.1) have been used to evaluate multicollinearity; all the variables have been examined and are within the appropriate ranges.
**Comment 4**: *Table 3 is not self-explanatory. The necessary content such as p value, r square, SE is not displayed. The way the authors describe the relationship is not easily understandable.*
**Author’ response***:* Thank you for the valuable comment. As correctly explained by reviewer, the statistical software output can be displayed as variable, coefficient, standard error, test statistics, sig. (p-value) and confidence interval. But these are relevant for the data analyzer to screen the variables but not for journal readers. The authors, in consultation with statisticians, have tried to make the statistical table as simple and understandable as possible for non-statisticians (avoiding the statistical jargon). Moreover, such type of presentation is space economical (minimizing the length of the table) and it is recommended by statistical authors as Monsalves et al.^8^

8. Monsalves MJ, Bangdiwala AS, Thabane A, Bangdiwala SI. LEVEL (Logical Explanations & Visualizations of Estimates in Linear mixed models): Recommendations for reporting multilevel data and analyses. BMC Med Res Methodol. 2020;20(1):1–9.

To make the table self explanatory, we have accepted the reviwers comment and corrected in the title on page 8.

**Comment 5**: *The authors should put references after ¨According to the CBNC implementation guideline; pregnant women should contact health professionals for antenatal, delivery and postnatal care services for skilled care.¨¨*

**Authors’ response**: Accepted and corrected on page 11.

**Reviewer two**

**Comment 1**: *Wording is not always clear like targeted site, illegible*

**Authors’ response**: Accepted and corrected on page 3.

**Comment 2**: *How you arrived or recruited your sample.*

**Authors’ response:** Kebeles were randomly selected, and then all postnatal mothers within six months of delivery, all health posts and health extension workers within the selected kebeles were recruited. It is well accepted and corrected in the document on page 3.

**Comment 3:** *It is also not clear if your the instrument you used for data collection was researcher developed and whether it has reliability and validity.*

**Authors’ response:** All study tools were author developed as described in data collection procedure section. Concerning validity and reliability of instruments, it has been checked and described in page 4 of the main document.

**Comment 4:** *It would be helpful too if there was more information about if only one person interviewed all the participants-the qualitative part or more than one person conducted these interviews. If there was more than one how was inter-rater reliability established.*

**Authors’ response**: Thank you for raising this very conceptual methodological issue. As clearly described in data collection procedure section of main document on page 4, the qualitative data were collected by two MpH holder facilitators. The data were collected using semi-structured questionnaire and tape recorded. The data coding, transcription and interpretation were made by the PI (one person). The issue of inter-rater reliability (IRR) preferably agreement will arise when them coding has been done two different personnel mainly for thematic analysis or grounded theory. But in our case, first, them coding were made by one person, second the data analysis technique required less interpretation and thirdly the authors used preformed frameworks for qualitative data analysis. In sum, the qualitative data analysis is less liable for inter-rater disagreement.

**Comment 5**: *What are your recommendations for next steps in this research? Clearly state that at the end*

**Authors’ response***:* The last sentence of discussion section on page 13 is a recommendation.

**Additional clarification**

Some of unfamiliar terms in the manuscript are due to the application/utilization of “standards for reporting implementation research (StaRI)” formats as the reporting guideline since it is an implementation science research.
