# Supplementary material for: Community-based newborn care intervention fidelity and its implementation drivers in South Wollo Zone, North-east Ethiopia
Source: PLOS Glob Public Health. 2023 Aug 11;3(8):e0001168. doi: 10.1371/journal.pgph.0001168 (PMC10420373; doi:10.1371/journal.pgph.0001168)
Supplement: Attachment — Submitted filename: Rebuttal letter.docx [file pgph.0001168.s002.docx]

March 19, 2023

Shela Hirani, PhD, IBCLC, RN
Academic Editor
PLOS Global Public Health

**Re**: Community-based newborn care intervention fidelity and its implementation drivers in South Wollo Zone, North-east Ethiopia (PGPH-D-22-01354R1)

Thank you for the constructive comments to improve our manuscript. We appreciate the time and effort the editor and reviewers have dedicated in reviewing our manuscript. We have revised the manuscript as per the comments and make a track change for those changes in the revised manuscript. We have also improved the quality of written English. . We believe the manuscript is now improved and suitable for publication at PLOS Global Public Health journal. We look forward hearing from you in due course.

Sincerely,

Asressie Molla Tessema (PhD), On behalf of all authors

**Here are the point-by-point response to the editor’s and reviewers’ comments and concerns.**

**Editor’s comment**

**Comment 1***: Please review your reference list to ensure that it is complete and correct. If you have cited papers that have been retracted, please include the rationale for doing so in the manuscript text, or remove these references and replace them with relevant current references. Any changes to the reference list should be mentioned in the rebuttal letter that accompanies your revised manuscript. If you need to cite a retracted article, indicate the article’s retracted status in the References list and also include a citation and full reference for the retraction notice.*

**Authors’ response**: We thank you very much for sharing your concerns. The reference completeness has been checked and corrected. Besides, all cited references has been checked and found to be not retracted from the journal.

**Reviewers’ comment**

**Reviewer one**

**Comment** 1: *Have the authors made all data underlying the findings in their manuscript fully available* 
**Authors’ response**: Accepted and corrected as indicated on page 14 of data availability section.

**Comment** 2: *PLOS Global Public Health does not copyedit accepted manuscripts, so the language in submitted articles must be clear, correct, and unambiguous. Any typographical or grammatical errors should be corrected at revision, so please note any specific errors here.*

**Authors’ response:** Thank you for the insight. All typological, grammatical and ambiguous issues were corrected.

**Comment** 3: *The addition to the methodology is very helpful. Only one question remains did the 12th grade graduates who had lived in the same kebeles who conducted the interview receive any training? Any inter-rater reliability done among the interviewers?*

**Authors’ response:** Thank you for indicating this important concept. Yes, training and test of reliability was conducted. Comments were well incorporated on pages 3- 4 of data collection section.
